# Resistance-guided therapy and integration of novel therapeutics are key to facilitating antimicrobial stewardship for sexually transmitted infections

**DOI:** 10.1017/ash.2026.10940

**Published:** 2026-10-29

**Authors:** Lao-Tzu Allan-Blitz, Jeffrey D. Klausner

**Affiliations:** 1 University of California Los Angeles, USA; 2 Keck School of Medicine University of Southern California, USA

Dear editor,

We read with great interest the article by *Mayi et al.* which discussed antimicrobial stewardship with respect to sexually transmitted infections (STIs).^1^ We commend the authors for highlighting this important topic. *Neisseria (N.) gonorrhoeae* is one of the top five antimicrobial resistant threats to public health^2^—with continued reports documenting the emergence of ceftriaxone resistance (our last line empiric therapy).^3,4^ Similarly, *Mycoplasma (M.) genitalium* is one of three organisms on the U.S. Centers for Disease Control and Prevention Antimicrobial Resistance Watch List.^2^ Thus, integrating antimicrobial stewardship into STI care and prevention is essential to mitigate the continued rise of drug resistance. However, two strategies central to facilitating antimicrobial stewardship within STIs have either been superficially mentioned or completely neglected: resistance-guided therapy and the incorporation of alternative empiric treatment options.

Resistance-guided therapy is the use of rapid molecular assays for identifying genetic determinants of antimicrobial resistance. For both *N. gonorrhoeae* and *M. genitalium*, such assays are commercially available and STI treatment guidelines permit their use. In *N. gonorrhoeae,* resistance-guided therapy using ciprofloxacin among strains with a wildtype codon 91 in the gyrase A gene has been shown to be >97% clinically efficacious across three studies.^5–7^ Real-world implementation of gyrase A genotyping led to a significant reduction in ceftriaxone use. Modeling demonstrated that rapid resistance testing can delay the emergence of ceftriaxone resistance.^8^ Across two clinical trials, more than 95% of *M. genitalium* infections were cured using macrolide-based resistance-guided therapy.^9,10^ That cure rate reflected a substantial improvement over the average 67% from other settings during the same time.^11^ Although no rapid molecular resistance test for either *N. gonorrhoeae* or *M. genitalium* are yet FDA-cleared, such tests have been CE marked in Europe and TGA approved in Australia. Further, a large clinical trial is underway evaluating the performance of three molecular resistance assays in *N. gonorrhoeae*, the results of which will support FDA 510(k) applications.

Importantly for *N. gonorrhoeae,* resistance-guided therapy carries additional benefits beyond antimicrobial stewardship. Ciprofloxacin is an oral therapy, obviating the need for intramuscular injection. That can improve patient comfort, but also permits electronic prescribing among asymptomatic patients, reducing the burden of returning to clinic for treatment. Access to and engagement with clinics may be challenging for many patients with gonorrhea due to the time required, perceived or experienced stigma, and structural factors limiting access to care. Finally, resistance-guided therapy may also increase the feasibility of expedited partner therapy as >97% of sex partners harbor *N. gonorrhoeae* strains with concordant resistance-determining genes.

Simultaneously, for the first time in decades the FDA has approved not one but two new anti-gonococcal agents: zoliflodacin and gepotidacin. Alternative treatment regimens are also increasingly under study for *M. genitalium* infection such as metronidazole, pristinamycin, sitafloxacin, and combinations of antibiotics, all of which have shown promise. How to integrate novel therapeutic options in a manner that most effectively reduces the selective pressure toward the emergence of resistance is precisely the purview of antimicrobial stewardship programs. For *N. gonorrhoeae,* modeling has indicated that reserving novel therapies until ceftriaxone resistance has emerged is the least effective strategy compared with combination therapy or equal allocation (randomly selecting one of the 2–3 available antimicrobials for any given case).^12,13^ However, clinical data and clear guidelines are needed.

Overall, antimicrobial resistance in bacterial STIs is an urgent public health threat. *Mayi et al.* have done an excellent job describing the importance of antimicrobial stewardship in general. However, to effectively mitigate the continued rise of drug-resistant pathogens, resistance-guided therapy and the thoughtful integration of novel therapeutics into existing antimicrobial stewardship efforts remain essential. Most notably, although there are many remaining questions, frontline providers and public health practitioners are finally becoming equipped with the necessary tools to meaningfully integrate antimicrobial stewardship into the field of sexual health.
